# History and development of national burden of disease assessment in Australia

**DOI:** 10.1186/s13690-020-00467-2

**Published:** 2020-09-29

**Authors:** Lynelle Moon, Michelle Gourley, John Goss, Miriam Lum On, Paula Laws, Anna Reynolds, Richard Juckes

**Affiliations:** 1 Health Group, Australian Institute of Health and Welfare, GPO Box 570, Canberra, ACT 2601 Australia; 2 Faculty of Health, University of Canberra, Canberra, Australia

**Keywords:** Burden, Disease, Australia, DALY, Method, Deaths, Risk factors

## Abstract

Australia’s 1996 national burden of disease (BoD) study was one of the first in the world and updates have continued over the following two decades with the fifth study now underway. The studies adapt the global framework most recently implemented by the Global Burden of Disease Study and the World Health Organization to suit Australia’s specific needs, producing estimates of fatal and non-fatal burden via the Disability Adjusted Life Year (DALY) metric, as well as attribution of the burden to many risk factors. Detailed Australian data are used with minimal reliance on modelling to fill data gaps. Comprehensive estimates are produced, including for the Indigenous population, for each of the eight states and territories, the five remoteness areas and five socioeconomic quintiles. A number of method developments have been made as part of these studies, including redistribution of deaths data and a detailed quality framework for describing the robustness of the underlying data and methods. Data and methods continue to be refined as part of the studies, and developments in global studies and other national studies are incorporated where appropriate.

## Background

Australia has been at the forefront of national burden of disease study development for more than 20 years. The Australian study for the 1996 reference year [1] was one of the first national-level burden of disease studies, along with those for Mexico, Mauritius and the Netherlands [2–4]. There have been periodic Australian studies completed since then, with the latest for the 2015 reference year [5].

All of the studies have been based at, or done in partnership with, the Australian Institute of Health and Welfare (AIHW), an independent government agency. Each study has been conducted in collaboration with many Australian experts, with the 2003 study in particular being conducted with the University of Queensland [6].

As a result of the initial studies, national and state/territory governments across Australia have become interested in using the burden of disease framework to help quantify health status and needs. There have also been advances in methods over the years and greater interest among health policy makers in information about burden of disease in particular population groups. This has stimulated the need for revised and continued Australian burden of disease studies to update and extend the initial efforts.

This paper describes the background of burden of disease work in Australia, a number of developments made (including use of direct data as much as possible, deaths redistribution, and a quality framework) and challenges faced over the years. The impact of the studies is also discussed.

## Development of Australian studies

**Fig. 1:**
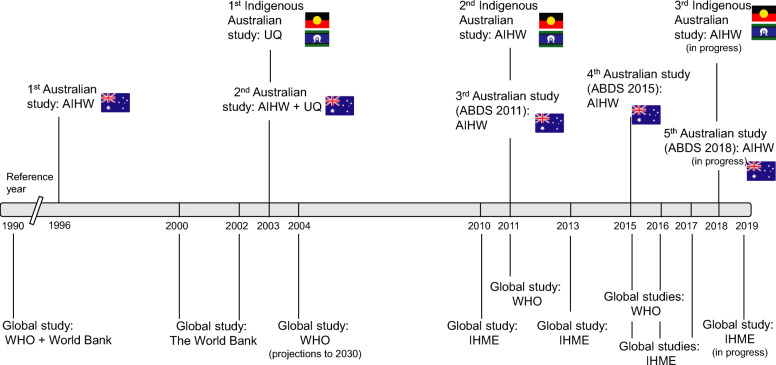
Global and national Australian studies

Australia has conducted four national burden of disease studies [1, 5–7], as well as two studies for Indigenous Australians [8, 9]. Figure 1 provides a summary of global [10–19] and national Australian studies [1, 5–9].

### First two national Australian studies: 1996 and 2003

The first study [1] used the 1996 reference year and was developed at the same time the Australian state of Victoria was conducting their own study [20, 21] with close collaboration between the two groups. These studies followed soon after the first global burden of disease study that had developed the DALY metric and quantified the global disease burden (and attribution to risk factors) for eight regions of the world [22]. The Australian study estimated DALYs lost due to 173 disease and injury groups and burden of disease attributable to 10 risk factors. It estimated DALY rates by socioeconomic quintiles and also estimated Disability adjusted life expectancy (later relabelled as Health-adjusted life expectancy (HALE)).

The second national Australian study was for the 2003 reference year [6], and was a joint project between the AIHW and the University of Queensland. This built on the analytical framework of the previous Australian study but included a number of improvements in methods (particularly around risk factor assessment where 14 risk factors were analysed including an estimate of the joint effect of all 14 risk factors). It used an updated version of the epidemiological modelling software DisMod (a commonly used tool in burden of disease studies to calculate missing epidemiological estimates, or to refine them [23]) which allowed for more accurate and consistent modelling epidemiological parameters. The 2003 study also included important extensions such as disease projections, analyses by remoteness and state-level burden of disease estimates. It was also the first time that a burden of disease study was conducted for the Indigenous population in Australia [9].

### ABDS 2011

There were significant revisions to methods for the third Australian study, which had become known as the Australian Burden of Disease Study (ABDS), covering the 2011 reference year [7]. Again estimates were produced for the Indigenous population [8]. The Global Burden of Disease Study (GBD) had undertaken a detailed review of methods used in previous global studies and published their first results in December 2012 [12] with DALY estimates for 2010. AIHW then undertook an assessment of the new global methods [24] to consider how it could best be used to meet Australia’s need for detailed estimates. From this review, the decision was made to undertake another national Australian study (rather than rely on the Australian estimates from GBD). It would use the updated global approach, using detailed Australian data sources and methods adapted to best suit the Australian context (including sub-national estimates). Upon commencement, the Australian study established a set of principles, including full transparency in data sources assumptions and methods, comparability with global methods where possible and the need to set up infrastructure for ongoing updates.

ABDS 2011 included 17 disease groups, around 200 specific diseases and 29 risk factors. The Years Lived with Disability (YLD) estimates were built up from approximately 320 disease sequelae which were further broken into 700 severity levels. Aspects of the GBD and World Health Organization (WHO) approaches were adopted including the simpler DALY, with YLD calculated using prevalence rather than incidence, and no age weighting or discounting; the new standard life table for Years of Life Lost (YLL) reflecting the theoretical maximum number of years to live at each age based on world-wide death data, equivalent for males and females [25]; and updated disability weights that reflected the general population’s valuation of different health states rather than being based on health experts’ judgement.

A number of new developments were also made with ABDS 2011 to reflect Australian specific needs. New diseases and risk factors were added to the list from the global studies (e.g. cancer of unknown primary site and sun exposure respectively). High-quality Australian data sources were used to derive estimates such as prevalence directly where possible (instead of modelling), and detailed methods for death redistribution were developed. Disease conceptual models for YLD calculations (the concepts used to estimate health loss from the disease or injury based on knowledge of disease pathways including sequelae, durations and severity distributions) were reviewed in conjunction with Australian experts, including data sources and assumptions. A quality scoring mechanism was developed and the ‘ABDS system’ was established (essentially a set of data input, processing, checking and output computer programs) to enable more streamlined updates to data in the future [26]. Some of these developments are discussed further below. Detailed advice was received from the project’s Advisory Group and Indigenous Advisory Group, along with a Jurisdictional Working Group with representatives from the 8 Australian states and territories, disease specific and risk factor expert panels and other national and international experts.

### ABDS 2015

The most recent Australian study covers the 2015 reference year [5]. Reflecting the importance that the ABDS evolves and incorporates improvements in both data and methods, it built on a number of extension projects completed since the 2011 study (discussed below). It also resulted in a more comprehensive list of diseases and risk factors, revised conceptual models for some diseases, improved data sources including greater use of linked hospital/deaths data, calculation of attributable burden by socioeconomic group and inclusion of HALE estimates. Data for 2003 and 2011 were updated using the revised methods, resulting in three time points, allowing for more detailed trend analysis. Detailed interactive data and visualisations were developed [27, 28]. Release of disease expenditure data using the same diseases and reference year was a significant development [29], enabling detailed comparison between the human cost and health system costs.

Significant differences exist between GBD’s estimates and those produced by ABDS. For example, GBD 2017 estimated there were 6.8% more YLLs and 22.3% more YLDs in Australia in 2015 than the ABDS 2015 study did. There were also large differences in Australian estimates between the 2 studies for specific diseases. For example, YLD estimates for coronary heart disease were 71% higher in ABDS 2015 than in GBD 2017 which was largely due to the use of Australian-specific data sources to estimate prevalence (hospitalisations and linked hospital and deaths data) whereas GBD used multiple sources from other countries to model prevalence.

## Specific Australian method developments

As discussed, there have been many developments during the life of the Australian national burden of disease studies. The early studies were among the first national studies to be undertaken worldwide. The latter studies applied country specific changes to the frameworks adopted at the international level including Indigenous and subnational estimates, new diseases and risk factors, and many modifications and data sources to best suit the Australian context. This section describes three of the key recent developments.

### Focus on direct use of data for estimate calculation

As a general principle ABDS 2011 and 2015 aimed to use high quality data to derive estimates directly where possible, rather than rely on modelling. The quality of the data was rigorously assessed before use, and built on many years of experience with monitoring systems for a particular disease group or expert knowledge of a particular data source. In the vast majority of cases, unit record data (person- or episode-level, rather than aggregate, data) were able to be used.

In some instances, a single, high quality data source was identified as being appropriate for the Australian context (such as the mortality data for the fatal burden estimates); in other cases, multiple data sources were used (such as using a primary data source to obtain a total sex-specific prevalence estimate and a secondary data source to obtain age distributions).

A general overview for ABDS 2015 (Table 1) shows the types of data sources used in the three main components of the study, along with other inputs and choices. For fatal burden calculations, the high-quality national death registration data were able to be used to calculate YLLs directly after redistribution of 10.5% of deaths that were coded to causes of death not appropriate for burden of disease analysis [30].

**Table 1:** Summary of inputs into calculation for ABDS 2015

	Fatal burden (YLL)	Non-fatal burden (YLD)	Risk factor attribution
**Main data sources**	*Number of deaths:* National Mortality Database	*Prevalence of disease:* Disease registers National Hospital Morbidity Database Linked hospital/deaths data Population health surveys Epidemiological studies	*Prevalence of risk factor:* Population health surveys Surveillance studies Disease registers National Hospital Morbidity Database National Mortality Database
**Other inputs**	Standard life table (GBD 2010)	Disability weights (GBD 2013)	Effect sizes/ relative risks and linked diseases (mostly GBD 2016)
**Key choices**	Redistribution method	Underlying conceptual model for each disease	Risk-outcome pairs Theoretical minimums

For the non-fatal component, prevalence (calculated at the sequelae-level), duration of disease and severity distribution are now based more on real data (rather than modelling) than in previous updates. This is closer to the fatal burden approach. As well as various disease registers (e.g. the cancer registry which has complete coverage as cancer is a notifiable disease), the national hospital database (used for diseases where treatment in hospital is common; principal and additional diagnoses are available) and representative population surveys, linked data are being more extensively used in each iteration of the study. This enables high quality prevalence estimates to be derived covering large portions of the population, such as for many cardiovascular diseases which either commonly are treated in hospital or are an underlying or associated cause of death. The ABDS 2015 made more extensive use of state-level linked hospital and deaths data, which were less progressed when conducting the ABDS 2011. Linked data were available for the two largest states (New South Wales and Victoria) in the ABDS 2015 (representing 57% of the population), whereas only linked data from one smaller state were used in the ABDS 2011. It is expected that close to national linked data will be available for future updates of the ABDS. Severity distributions were based on Australian data where possible (such as using Australian epidemiological studies, national surveys or hospitalisation data) [26].

For the risk factor analysis, modelling is still required for many aspects of the analysis. However, in some cases it was possible to use direct evidence to quantify the proportion of diseases resulting from the risk factor. This was the case for homicide from intimate partner violence, for various infectious diseases and for some other risk–outcome pairs [30].

### Redistribution of deaths

As part of ABDS 2011 and ABDS 2015, methods were developed to redistribute causes of death not considered appropriate for burden of disease analysis (10.5% of 2015 deaths were redistributed in ABDS 2015^30^) [30]. These methods use Australian evidence in three main ways. The first two methods use empirical evidence, with these able to be used in 86% of redistributed causes of death in ABDS 2015, while the third is only used when neither of the others are possible. In many cases the redistribution results in a more specific cause of death still within the original disease group. Redistribution within a disease group accounted for 43.1% of the redistributed deaths for the 2015 estimates. An example is unspecified cancers which are allocated to specific cancer causes of death.

The first method uses direct evidence for the particular deaths identified for redistribution, and it is considered the best method to use when suitable data are available. Information is obtained through data linkage studies or from sources other than the National Mortality Database to ascertain probabilities of a more plausible cause of death. Direct evidence was able to be used for 39.4% of the redistributed causes for the 2015 estimates, and partially for another 8.4%.

As an example, deaths with an original cause of death of unknown primary cancer were redistributed to account for some of these being true cancers of unknown primary site and some being coded to that cause due to insufficient information being available on the death certificate to accurately assign the correct cancer site [31]. We were able to use cancer registry data from two Australian states that included detailed data on all cancers over a 5 year period from the cancer incidence notification process (such as pathology or hospital admission data) linked to death registration data. This provided rich information on the individuals’ health profile to further clarify what may be the most appropriate cause of death. From these data, it was found that between 60.4–74.9% of deaths with this cause of death were ‘true’ cancers of unknown primary site, while the remaining were better allocated to other cancer causes of death.

The second method uses the extra information available in Australia’s multiple causes of death data, and is termed the Indirect multiple causes of death (MCOD) method. It uses the pattern of the underlying causes of death (UCOD) where the cause identified for redistribution was mentioned as an associated cause of death. The corresponding UCODs and their proportional distribution provide the redistribution algorithm. For example, for deaths with an underlying cause of septicaemia, all deaths that mentioned septicaemia as an associated cause of death were identified. The corresponding proportional distribution of UCODs of these records reflect a pattern of underlying causes of death for deaths that involved septicaemia. The septicaemia deaths needing to be redistributed were then reallocated using this proportional distribution. The indirect MCOD method was applied where the redistribution was for one of the most commonly occurring causes of death and no direct evidence was available. It was used for 37.8% of redistributed deaths in ABDS 2015, and partially for another 8.4%.

The final method used was proportional redistribution to specified target causes(s). This method reassigns deaths across a range of target causes selected according to: the existing distribution of underlying cause of death within that disease group; or expert advice; or the GBD redistribution algorithms. This method has the advantage of being a conceptually simple approach but may not be well customised to a particular cause of death. Because of this, it was considered appropriate only for low-volume redistribution causes where direct evidence was not available or where the indirect MCOD method was not suitable. It was only used for 14.5% of redistributed causes in 2015.

The impact of using these new redistribution methods was compared to the GBD methods using deaths occurring in 2010. Using the Australian redistribution methods, approximately 10% of deaths were identified for redistribution in that year. This compares with 18% of Australian deaths that were identified using GBD algorithms. The difference is largely due to the disease list used: some of the causes of death that were redistributed in the GBD study were directly allocated to a specified disease in the Australian study [7].

### Quality framework

Uncertainty intervals (a range of values that reflect the uncertainty in the estimate which is likely to include the correct estimate) have not been included in the ABDS. Such estimation of uncertainty would need to take into account the complex analysis and manipulation needed to align the input data to the preferred epidemiological variables, disease definitions, population and time period. This would require a combination of assumptions, models and judgments. Measures of uncertainty would need to take into account uncertainties in both the data (such as standard errors from surveys and misalignment with our preferred case definition) and the models and transformations (such as estimating prevalence from incidence and estimating sub-national estimates). The amount of work required to develop a reasonable and defensible method of uncertainty estimation that could be used across all parts of the ABDS was not within the resources of the project.

Instead, a quality framework was developed to describe the reliability and limitations of the estimates, such as which patterns and differences were likely to be genuine, and which could be influenced by uncertainties in the data or methods that made them less reliable [26, 30]. It includes a quality index (described below) and, to assist in understanding the ratings in the index, a short text description of the main data sources and transformations needed.

The index has two dimensions covering a) the relevance of the underlying epidemiological **data** and b) the **methods** used to transform that data into a form required for the analysis. The final index also took into account the contribution of the underlying data to the overall estimate. For example, a particular data source might have contributed a large proportion of the overall YLD for a single disease, while another might have only contributed a small proportion. A scoring system was developed, tested and applied to 194 diseases for YLD calculations and 18 risk factors in the most recent ABDS [30]. No ratings were provided for YLL estimates as the mortality data were considered to be comprehensive and relevant with little or no transformation required other than the redistribution of a small proportion of deaths.

Most diseases (60.8%) and risk factors (72.3%) were based on data that was ‘relevant’ or ‘highly relevant’ (Table 2). Around half of the diseases (48.5%) and risk factors (50%) required little or no data transformation. At the other end of the spectrum, only a small number of disease or risk factor estimates were based on questionable data or methods.

**Table 2:** Summary of quality index assessment, ABDS 2015

	YLD		Risk factors	
	Number of diseases	% of diseases	Number of risk factors	% of risk factors
*Dimension 1 - data*				
A - Highly relevant—estimate was derived from comprehensive and highly relevant data	73	37.6	3	16.7
B - Relevant	45	23.2	10	55.6
C - Moderately relevant/accurate—estimate was derived from reasonably comprehensive and relevant data	45	23.2	3	16.7
D - Somewhat relevant	29	14.9	1	5.6
E - Questionable relevance—use with caution, as estimate was derived from less comprehensive or relevant data	2	1.0	1	5.6
**Total**	194	100.0	18	100.0
*Dimension 2 - methods*				
A - Highly accurate—no data transformation was required	56	28.9	2	11.1
B - Accurate	38	19.6	7	38.9
C - Moderately accurate—moderate transformations required, taking into account known trends in the underlying data, such as over time or age-distributions	61	31.4	6	33.3
D - Somewhat accurate	35	18.0	1	5.6
E - Questionable accuracy—use with caution, moderate transformations required with trends unknown or unaccounted for	4	2.1	0	0.0
Unable to be assigned	0	0.0	2	11.1
**Total**	**194**	**100.0**	**18**	**100.0**

## Use and impact

The ABDS is being increasingly used for population health monitoring, policy decisions and in research. Each of the four national burden of disease studies have been funded by the Australian Government Department of Health, indicating the importance of this resource to policy development. Also at the national level, portfolio agencies outside health have used the burden of disease analysis [32]. In addition, all state and territory health departments have used the jurisdictional estimates for their policy and planning and some included it in their main health reports [33–35] or as the basis for their own burden of disease estimates [36, 37].

The peer-reviewed literature shows that, since the release of ABDS 2011, there has been an incremental increase in papers citing the Study. This has occurred despite the fact that ABDS publications are all formally classed as ‘grey’ literature and are therefore difficult to locate using the reference databases. The majority of research articles that cite ABDS use it as evidence for supporting further clinical or methodological research projects [38]. A number of articles are advocacy related opinion pieces [39, 40] commenting on the discrepancy between health funding and disease burden, or aim to promote areas of health burden often overlooked [41–43]. The majority of research articles that cite ABDS are disease specific, with the most common being related to cardiovascular disease and cancer [44]. Almost all articles are Australian based, although ABDS 2011 estimates were used in a multi-country study on sex differences in mortality due to stroke [45].

A stakeholder survey was conducted in 2018 to assess the use and impact of ABDS 2011. A questionnaire was distributed to government organisations, universities, research organisations, not for profits, medical colleges and via Twitter. Half of the 293 respondents had used ABDS 2011 products. While this was not a random survey, it does provide some contextual information on how the products were being used. The three main uses reported by participants were: as background literature (60%), citing the results (44%) and in policy development (30%). About 37% of ABDS users said the Study had already contributed to policy, health planning and funding, and a further 13% stated that it would be used for this purpose in the future. A formal assessment of the use and impact of ABDS 2015 (released in 2019) has not yet been undertaken.

Between ABDS 2011 and 2015, a number of extension projects were conducted by the project team at AIHW, to undertake more in-depth analysis on particular diseases including cancer [31], chronic respiratory conditions [46], musculoskeletal conditions [47] and lower limb amputations [48]; or particular risk factors including overweight and obesity [49], physical inactivity [50], intimate partner violence [51] and alcohol and illicit drug use [52], and since the release of ABDS 2015 there has been an extension project on tobacco use published [53]. Other extension projects further developed methods which have subsequently been incorporated into the ABDS system such as diseases as risks [54], vascular risk factors for dementia [55] and HALE [56]. A number of the risk factor reports, such as those on overweight and obesity [49], physical inactivity [50], and tobacco use [53] included scenario modelling which analysed the potential future burden under various alternative risk factor trends. For example, 14% of the expected disease burden due to overweight and obesity in 2020 would have been avoided if the population at risk in 2011 reduced their body mass index by 1 and these rates were maintained to 2020.

## Current focus and remaining challenges

The main current focus for the ABDS is to produce another update, this time for the 2018 reference year. This study is expected to be released in 2021 and will include further improvements in data sources, such as expanded coverage of linked data and the expected filling of some key data gaps. In order to complete the many components of a burden of disease study and to ensure consistency across the components, the ABDS team members meet regularly and all have multiple roles such as coordinating one or more disease groups as well as working on outputs and data visualisations. With the addition of a fourth year of data, the ABDS 2018 will include 5.4 million estimates. In addition, an Indigenous Burden of Disease Study is being undertaken for 2018. There is also an extension project using HALE underway to provide further insight into whether longer lives are necessarily healthier lives with Australia’s ever-increasing life expectancy.

The key remaining data and methods challenges relate to the non-fatal component of the study. The first is the method used to derive the disability weights which remain the subject of international discussion and debate [57–60]. The set of disability weights used in the most recent version of the ABDS come from the GBD 2013. The weights are based on surveys of populations in a number of countries as well as on an internet survey [61] and are still being used in the most recent GBD studies. Analysis of the results suggests there was little variation between countries in these valuations. However, to date, no specific validation in the Australian context has been able to be undertaken. Other national studies have developed their own disability weights [62] or have adjusted the GBD weights to better suit their purposes or to bring in extra data [13, 63]. Some work has also been undertaken by academics to calculate alternative patient-based disability weights for injury diagnoses using self-reported data from a number of countries including Australia [64].

Another area where improvements could be made relates to the severity distributions (which represent the proportion of people for a particular disease by levels of severity), given the significant impact these can have on the final estimates [65, 66]. The ABDS was able to use Australian data for some severity distributions (including cancers and a number of injuries, musculoskeletal conditions, vision-related disorders, respiratory diseases and neurological diseases [26]), but relied on the GBD distributions for others. For many of these, the GBD had used data from the United States and Australian surveys, making it likely they are appropriate in many cases, though others may be improved with Australian-specific data.

Finally, there remain particular diseases and risk factors where the prevalence data are less accurate than is desirable. These include some diseases and a risk factor which received the lowest quality score [30]: Benign and uncertain brain tumours, Parkinson disease and Low bone mineral density. At a disease group level, two disease groups that account for a large proportion of the YLD burden also have limitations: the mental health prevalence data were 8 years old for the 2015 estimates, and the musculoskeletal self-reported data are known to overlap with injury data. The situation for these disease groups will improve with a new mental health survey expected to be run in the near future and methodological work initiated to better separate musculoskeletal and injury sequelae.

## Conclusion

Australia now has well established and regular national burden of disease studies that use the framework of the latest international studies adapted to the Australian context. The methods and data used in these studies continue to improve with each new update. As a country with high-quality health information, the studies are able to use very relevant unit record data with minimal need for modelling. The ABDS has been established as a national asset and is undertaken within an independent government data agency, the AIHW, providing access to high-quality data, a rigorous methodological and statistical review process, access to numerous disease-specific expert advisory groups, and a good level of stability in staff with expertise in burden of disease analysis and methods.

The ABDS is used extensively within Australia for various health monitoring, policy and research needs. In addition, Australia also uses global studies to enable valid comparisons between Australia’s burden of disease and that in other countries. This mirrors our use of other international data, such as those produced by the OECD. While the Australian data that are part of the international dataset may not exactly match that preferred for use within Australia, consistent definitions have been used to enable valid comparisons across countries.

The ABDS continues to use various inputs from GBD (such as the disability weights, reference life table and severity distributions for a number of conditions), but the estimates from the Australian national study are strongly preferred for Australia’s policy and health monitoring needs, due to being based on detailed Australian data and methods developed for the Australian context.

There remain a number of opportunities for further use of the estimates as well as further methods development. The database underlying the Australian estimates is very detailed and can be used as the basis for more in-depth analyses, such as of age/sex groups or subnational groups, or further modelling for policy purposes, such as scenario modelling or projections. Work has also commenced on a project in conjunction with academic researchers on how the multiple causes of death data can potentially be used to improve future YLL estimates, rather than just relying on the single underlying cause of death.

The AIHW is continuing to build relationships with other countries undertaking burden of disease work to share learnings, continue to improve methods and contribute to the body of knowledge and international expertise in this area.

## Data Availability

Summary data tables and data visualisations from the 2015 ABDS are available at https://www.aihw.gov.au/reports-data/health-conditions-disability-deaths/burden-of-disease/data. Detailed data from ABDS 2015 (including 2003, 2011 and 2015 data) are available upon request on a cost recovery basis from the AIHW burden of disease team: email burdenofdisease@aihw.gov.au; address: GPO Box 570 Canberra ACT 2601 Australia.

## Abbreviations

ABDS: Australian Burden of Disease Study
AIHW: Australian Institute of Health and Welfare
DALY: Disability-Adjusted Life Year
GBD: Global Burden of Disease
HALE: Health-adjusted life expectancy
MCOD: multiple cause of death
UCOD: underlying cause of death
YLD: Years lived with disability
YLL: Years of life lost
